# Natural Minerals Coated by Biopolymer Chitosan: Synthesis, Physicochemical, and Adsorption Properties

**DOI:** 10.1186/s11671-016-1696-y

**Published:** 2016-11-08

**Authors:** T. M. Budnyak, E. S. Yanovska, O. Yu. Kichkiruk, D. Sternik, V. A. Tertykh

**Affiliations:** 1 Chuiko Institute of Surface Chemistry of National Academy of Sciences of Ukraine, 17 General Naumov Str., 03164 Kyiv, Ukraine; 2 Taras Shevchenko National University of Kyiv, 62a Volodymyrska Str., 01033 Kyiv, Ukraine; 3 Zhytomyr Ivan Franko State University, 42 Pushkina Str., Zhytomyr, Ukraine; 4 Maria Curie-Skłodowska University, 2 Maria Curie Sklodowska Sq.,, 20-031 Lublin, Poland

**Keywords:** Adsorption, Chitosan, Composite, Heavy metals, Calorimetry, Thermal analysis

## Abstract

Natural minerals are widely used in treatment technologies as mineral fertilizer, food additive in animal husbandry, and cosmetics because they combine valuable ion-exchanging and adsorption properties together with unique physicochemical and medical properties. Saponite (saponite clay) of the Ukrainian Podillya refers to the class of bentonites, a subclass of layered magnesium silicate montmorillonite. Clinoptilolits are aluminosilicates with carcase structure. In our work, we have coated biopolymer chitosan on the surfaces of natural minerals of Ukrainian origin — Podilsky saponite and Sokyrnitsky clinoptilolite. Chitosan mineral composites have been obtained by crosslinking of adsorbed biopolymer ﻿on﻿ saponite and clinoptilolite surface with glutaraldehyde. The obtained composites have been characterized by the physicochemical methods such as thermogravimetric/differential thermal analyses (DTA, DTG, TG), differential scanning calorimetry, mass analysis, nitrogen adsorption/desorption isotherms, scanning electron microscopy (SEM), and Fourier transform infrared (FTIR) spectroscopy to determine possible interactions between the silica and chitosan molecule. The adsorption of microquantities of cations Cu(II), Zn(II), Fe(III), Cd(II), and Pb(II) by the obtained composites and the initial natural minerals has been studied from aqueous solutions. The sorption capacities and kinetic adsorption characteristics of the adsorbents were estimated. It was found that the obtained results have shown that the ability of chitosan to coordinate heavy metal ions Zn(II), Cu(II), Cd(II), and Fe(III) is less or equal to the ability to retain ions of these metals in the pores of minerals without forming chemical bonds.

## Background

Application of chitinous products in wastewater treatment has received considerable attention in recent years in the literature [1–8]. In particular, the development of chitosan-based materials as useful adsorbent polymeric matrices is an expanding field in the area of adsorption science [9]. Chitosan is a type of natural polyaminosaccharide, obtained by deacetylation of chitin [10], which is a polysaccharide consisting predominantly of unbranched chains of *β*-(1→4)-2-acetoamido-2-deoxy-*D*-glucose [11]. Composites based on chitosan are economically feasible because they are easy to prepare and involve inexpensive chemical reagents [11]. Recently, chitosan composites have been developed to adsorb heavy metals and dyes from wastewater [10, 12–15].

Chitosan composites have been proven to have better adsorption capacity and resistance to acidic environment [11]. Various methods of preparation of hybrid materials based on inorganic materials and polysaccharides such as chitin [1–8] and chitosan for different applications have been studied [9, 11, 16–18]. Different kinds of substances have been used to form composite with chitosan such as silica, montmorillonite, polyurethane, activated clay, bentonite, polyvinyl alcohol, polyvinyl chloride, kaolinite, oil palm ash, perlite, and magnetite [19–23]. Although such minerals possess high adsorption capabilities, the modification of their structure can successfully improve their capabilities. In work [24], chitosan/attapulgite composites are applied as an adsorbent for the removal of chromium and iron ions from aqueous solution of both single and binary systems. Attapulgite is a hydrated octahedral-layered magnesium aluminum silicate mineral with large surface area, excellent chemical stability, and strong adsorption. Equilibrium data were well described by the Freundlich isotherm models, indicating multilayer adsorption for Cr(III) and Fe(III) onto composites. Kinetic experiments showed that composites offered fast kinetics for adsorption of Cr(III) and Fe(III), and the diffusion-controlled process as the essential adsorption rate-controlling step was also proposed. Moreover, the initial adsorption rates of Cr(III) were faster than that of Fe(III) with the increase of temperature and initial concentrations. The thermodynamic analysis presented the endothermic, spontaneous, and entropy gained nature of the process [24].

The removal of nickel (II) from the aqueous solutions through adsorption on to biopolymer sorbents, such as calcium alginate, chitosan-coated calcium alginate, and chitosan-coated silica, was studied using equilibrium batch and column flow techniques. According to the study, the maximum monolayer adsorption capacity of calcium alginate, chitosan-coated calcium alginate, and chitosan-coated silica, as obtained from the Langmuir adsorption isotherm, was found to be 310.4, 222.2, and 254.3 mg/g, respectively [25].

Polymer/montmorillonite nanocomposites have improved properties such as excellent mechanical properties, thermal stability, gas barrier, and flame retardation in comparison to conventional composites. The isomorphous substitutions of Al^3+^ for Si^4+^ in the tetrahedral layer and Mg^2+^ for Al^3+^ in the octahedral layer have resulted in a negatively charged surface on montmorillonite. With these structural characteristics, montmorillonite has excellent sorption properties and possesses available sorption sites within its interlayer space as well as large surface area and more narrow channels inside. Produced chitosan coated montmorillonite for the removal of Cr(VI) [11].

This work describes the synthesis of the composite material based on chitosan and natural minerals clinoptilolite and saponite, for their use as a biosorbents. Obtained composites were characterized by physicochemical methods, such as thermal analysis and textural properties. Adsorption properties of the obtained hybrid material were studied with respect to highly toxic heavy metals: cadmium(II), lead(II), copper(II), zinc(II), and iron(III), which are common contaminants of industrial wastewaters. Conditions connected with the optimum pH value of the medium, interaction time, and adsorption capacity were studied.

## Experimental part

### Materials

Sokyrnitskiy clinoptilolite of Ukrainian Zakarpattya has the general formula (Ca,Na,K_2_)Al_2_Si_7_O_18_·6H_2_O, chemical content (in mass %): SiO_2_—76.07; Al_2_O_3_—12.4; K_2_O—2.80; CaO—2.09; Na_2_O—2.05; Fe_2_O_3_—0.90; FeO—0.76; TiO_2_—0.19; P_2_O_5_—0.12; MgO—0.07; MnO—0.07; SO_3_—0.08. Saponite of Ukrainian Podillya has the general formula (Ca_0.5_,Na)_0.33_(Mg,Fe)_3_(Si,Al)_4_O_10_(OH)_2_·4H_2_O. Chitosan is originally from shrimps, Sigma-Aldrich, No. 417963, molecular weight from 190,000 to 370,000 Da, degree of deacetylation — not less than 75%, and solubility 10 mg/ml. All chemicals are purchased from Sigma-Aldrich were of reagent grade.

### Methods

- *Composites chitosan-saponite and chitosan-clinoptilolite* were obtained by impregnation 20 g of minerals (saponite and clinoptilolite) by 285 ml of chitosan solution with a concentration of 7 mg/ml in acetic acid (pH 2.6). The mixture was put in flat-bottom flask and mixed by the magnetic stirrer MM-5 for 2 h. The obtained substance was dried at 50 °C. The obtained composites were placed in 12.5 ml of 0.25% solution of glutaraldehyde in water and heated at 50 °C for 2 h. Such quantity of glutaraldehyde is proper for crosslinking of 5% of accessible amino groups of polymer. The crosslinked chitosan on the surface of the minerals were washed with distilled water and dried at 50 °C. Thus, based on the theoretical mass ratio, the obtained organic and mineral components of the composite was chitosan:silica = 1:10 [15].
- *Buffer solutions* with pH 1.0 prepared from the standard titrimetric substance of HCl acid, pH 2.5, and 5.0 from glacial acetic acid, and pH 8.0 were prepared from 17 ml of 1 M acetic acid and 5 ml of 25% ammonia solution and adding distilled water up to 1 l. The pH values of all buffer solutions were controlled by a pH meter.
- *FTIR spectra* of the samples of the initial chitosan and reaction products were recorded using an IR spectrometer with Fourier transformation (Thermo Nicolet Nexus FT-IR, USA). For this purpose, the samples were ground in an agate mortar and pressed with KBr.
- *Thermal analysis.* Thermal analysis was carried out on a STA 449 Jupiter F1, Netzsch (Germany) under the following operational conditions: heating rate of 10 °C min^−1^, a dynamic atmosphere of synthetic air (50 ml min^−1^), temperature range of 30–950 °C, sample mass ~18 mg, and sensor thermocouple type S TG-DSC. As a reference, empty Al_2_O_3_ crucible was used. The gaseous products emitted during decomposition of materials were analyzed by FTIR spectrometer Brucker (Germany) and by QMS 403C Aeölos (Germany) coupling online to the STA instrument. The QMS data were gathered in the range of from 10 to 160 amu. The FTIR spectra were recorded in the spectral range of 600–4000 cm^−1^ with 16 scans per spectrum at a resolution of 4 cm^−1^.
- *Surface area and average pore diameter analysis.* The specific surface area and the average pore diameter of the composite were determined with the BET instrument ASAP 2405 (Micromeritics Instrument Co., USA). The isotherm plots were used to calculate the specific surface area and the average pore diameter of chitosan-silica composite.
- *Surface morphology analysis.* The surface morphology of chitosan–silica composite was observed by using a scanning electron microscope (SEM, LEO 1430VP, Carl Zeiss, Germany).

The investigations of adsorption properties of the obtained composite with respect to zinc, copper, cadmium, lead, and iron were carried out in the static mode with periodic hand-stirring. For that, the sample of 0.1 g of synthesized adsorbent was contacted with 25 ml of solutions at different concentrations of salts: Zn(NO_3_)_2_·6H_2_O, CuCl_2_·2H_2_O, Cd(NO_3_)_2_·4H_2_O, Pb(NO_3_)_2_, FeCl_3_, which were prepared according to [26]. Determination of the equilibrium concentration of the metals was carried out by atomic absorption using a flaming atomic absorption spectrophotometer “Saturn” (Ukraine) in a “air-propane-butane” flame mixture.

### Calculations

The adsorption capacity (*q*_*e*_) was calculated using the formula:

$$ {q}_e = \left({c}_0\hbox{--} {c}_e\right)V\ /\ m; $$

the degree of adsorption (*R*) was calculated using the formula:

$$ R = \left({c}_{\mathrm{ads}}/{c}_0\right)\cdotp 100\% = \left({c}_0-{c}_e\right)/{c}_0\cdotp 100\%, $$

where *c*_0_ is the concentration of initial solution, *c*_e_ is the equilibrium concentration of metal, *V* is the volume of equilibrium solution, and *m* is the mass of adsorbent.

## Results and Discussion

### Physicochemical Characteristics of the Composite

**Fig. 1:**
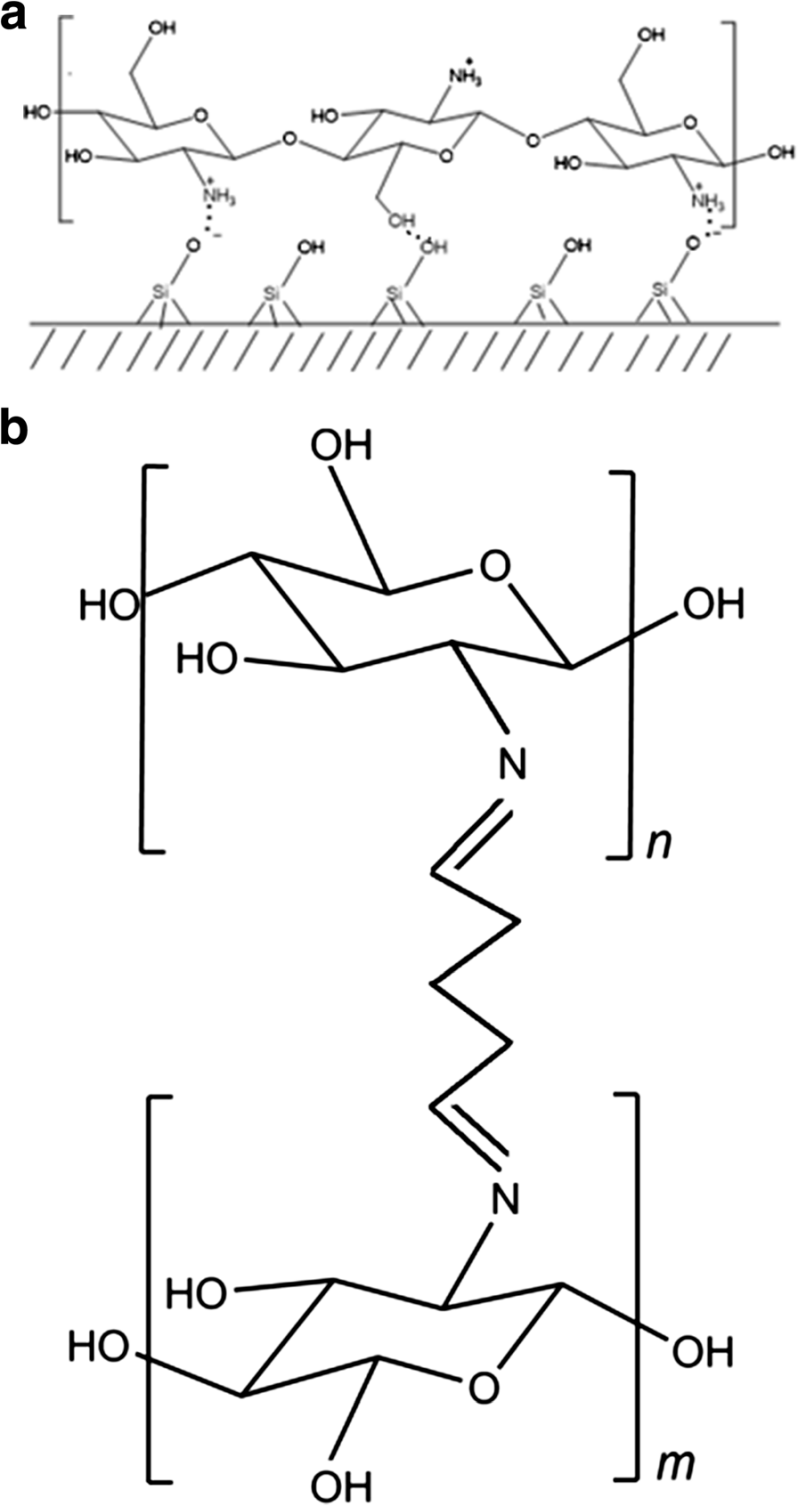
The schemes of the structure of chitosan-mineral composites **(a)** and crosslinking of surface layer of adsorbed polymer (**b**)

Chitosan has a high affinity to the surface of silica-based minerals due to the interaction between part of protonated amino groups of polymer and dissociated hydroxyl groups of silica, which are formed in aqueous solution [15]. Thus, the mechanism of the chitosan interaction with the selected minerals is due to the electrostatic interaction as well as hydrogen binding. The scheme of structure of chitosan mineral composites is presented in Fig. 1.

**Fig. 2:**
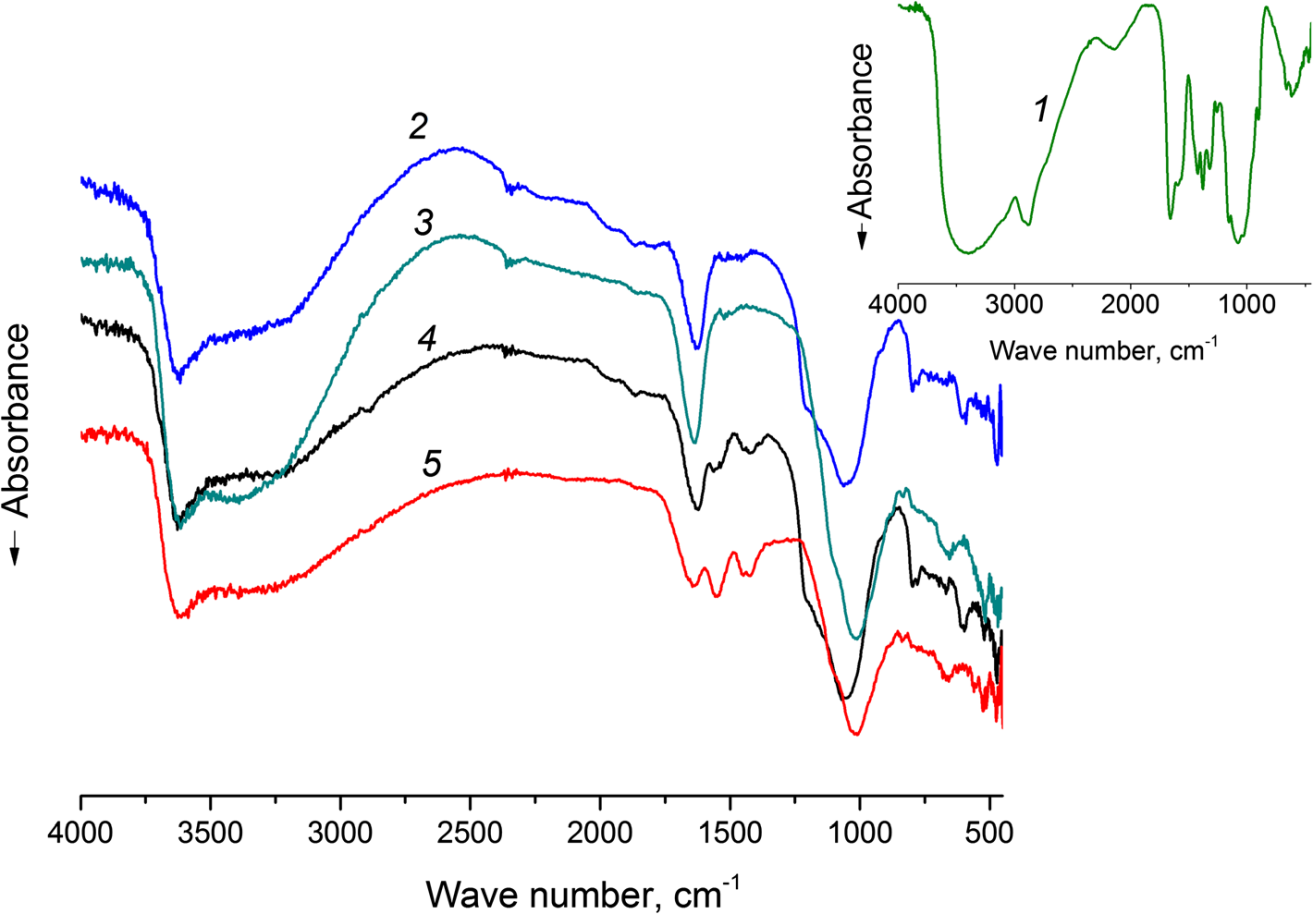
FTIR spectra of chitosan (*1*), clinoptilolite (*2*), saponite (*3*) and chitosan-clinoptilolite (*4*), and chitosan-saponite composite (*5*)

In order to ascertain the immobilization of chitosan onto the surface of minerals, FTIR spectroscopy was employed to characterize initial chitosan, clinoptilolite, saponite, and synthesized composites (Fig. 2). In the FTIR spectrum of chitosan (Fig. 2 (1)), the band at 3429 cm^−1^ corresponds to the stretching vibrations O–H of hydroxyl groups bound with carbon atoms. Intensive absorption bands at 2800–3000 cm^−1^ are observed due to the C–H stretching vibrations. The band at 1580 cm^−1^ corresponds to the deformation vibrations of –NH_2_, 1420 and 1380 cm^−1^ for C–H binding vibrations, 1310 cm^−1^ for asymmetric C–O–C stretching vibrations, and 1080 cm^−1^ for C–O stretching vibration of CH–OH.

The FTIR spectrum of the synthesized composites (Fig. 2 (4 and 5)) has shown a shift of the band 1530 cm^-1^ of –NH_2_ deformation vibrations in comparison with the spectrum of the initial chitosan. An intensive absorbance at 1090 and 1000 cm^−1^ represents the Si–O stretching vibrations. Absorbance band at 610 and 660 cm^−1^ represents to stretching vibrations of Si–O and shifted in comparison with the FTIR-spectra of initial minerals. Absorbance bands at 556 and 463 cm^−1^ and 518 and 466 cm^−1^ represent the deformation vibrations of Al–O–Si and Si–O–Si in chitosan-clinoptilolite and chitosan-saponite, respectively. It was observed that the characteristic bands at 1633 and 1645 cm^−1^ at the FTIR-spectra of chitosan-clinoptilolite and chitosan-saponite, respectively, describe azomethine bonds C=N, formed after glutaraldehyde treatment [27].

The influence of polymeric coating on thermal properties of mineral surfaces was studied by conducting DSC-MS analysis. Applying of these methods of investigations were also conducted in order to determine the mass ratio of chitosan coating on the mineral surfaces.

**Fig. 3:**
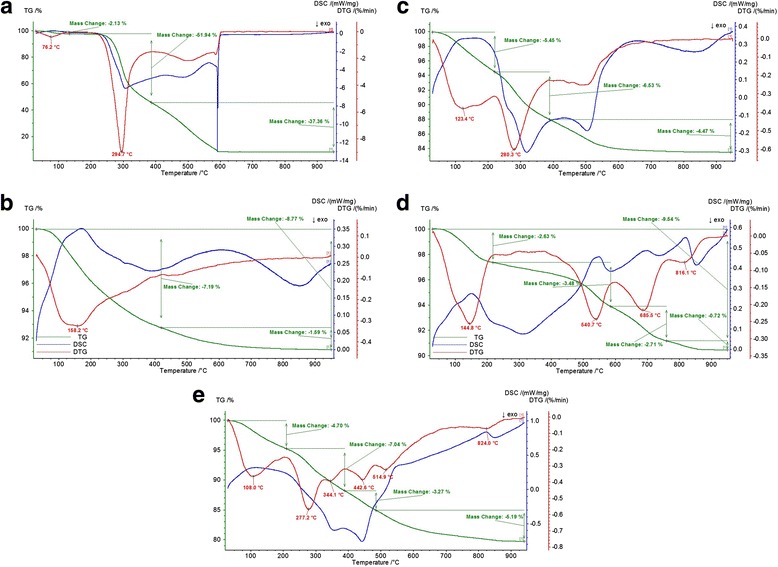
TG, DTG, and DSC curves of chitosan (**a**), clinoptilolite **(b)**, chitosan-clinoptilolite composite (**c**), saponite (**d**), and chitosan-saponite composite (**e**)

For the TG-curve of chitosan (Fig. 3a), two decomposition temperatures can be found. The initial weight loss of 11% from room temperature (30 °C) up to 190 °C corresponds to the release of adsorbed water [21]. The second recorded decomposition region (190–1000 °C) completely applies to the weight loss of chitosan. Figure 3b, c presents the TG, DTG, and DSC curves of pure clinoptilolite and saponite.

Comparing the thermogravimetric curves of chitosan-clinoptilolite and chitosan-saponite composites (with the curves of the initial chitosan and pure minerals; Fig. 3d, e), one could observe that the maximum of each decomposition region of composites was observed at lower temperatures than for similar process of the pure minerals. For instance, coated clinoptilolite and saponite begin lost the water at *T*_max_ 123 and 108 °C, when pure minerals – at 159 and 145 °C, respectively (Table 1). The main decomposition of the composite materials occurred at *T*_max_ 280 and 277 °C when pure clinoptilolite and saponite did not show decomposition at this temperatures, in contrast with the native chitosan, which is characterized by loss of more than 50% at *T*_max_ 295 °C. Thus, the coated minerals are able to lose water faster than pure minerals and the temperature of the decomposition of polymer in composition of hybrid materials decreased by 15 °C (5 %) for chitosan-clinoptilolite composite and by 18 °C (6 %) for chitosan-saponite composite.

**Table 1:** The comparison of thermal characteristics of initial chitosan, clinoptilolite, saponite, and synthesized composites of partially crosslinked chitosan and minerals

Material	*T*_max_, °C (DTG)	∆*m*, % (TG)	∆*m*_total_, % (TG)	*T*_max_, °C (DSC)	*m*/*z* (MS)
Chitosan	76 295 500	2.13 51.94 37.67	93	300 400	12 16 18 30 44
Partially crosslinked chitosan-clinoptilolite	123 280 500	5.45 6.53 4.47	16.5	320 515 850	12 16 18 44
Clinoptilolite	159	7.19 1.59	8.77	400 860	16 18
Partially crosslinked chitosan-saponite	108 277 344 443 515 824	4.70 7.04 3.27 5.19	20	350 450 510 860	12 16 18 22 30 41 44
Saponite	145 541 686 816	2.63 3.48 0.72	9.5	220 320 590 730 860	12 16 18 44

Comparing the results of thermogravimetric analysis for the initial and obtained composites, it was confirmed that all involved polymers to the reaction were successfully introduced to the hybrid materials. Thus, each composite contains 10 % polymer and 90 % of the mineral part (91 mg/g of chitosan).

**Fig. 4:**
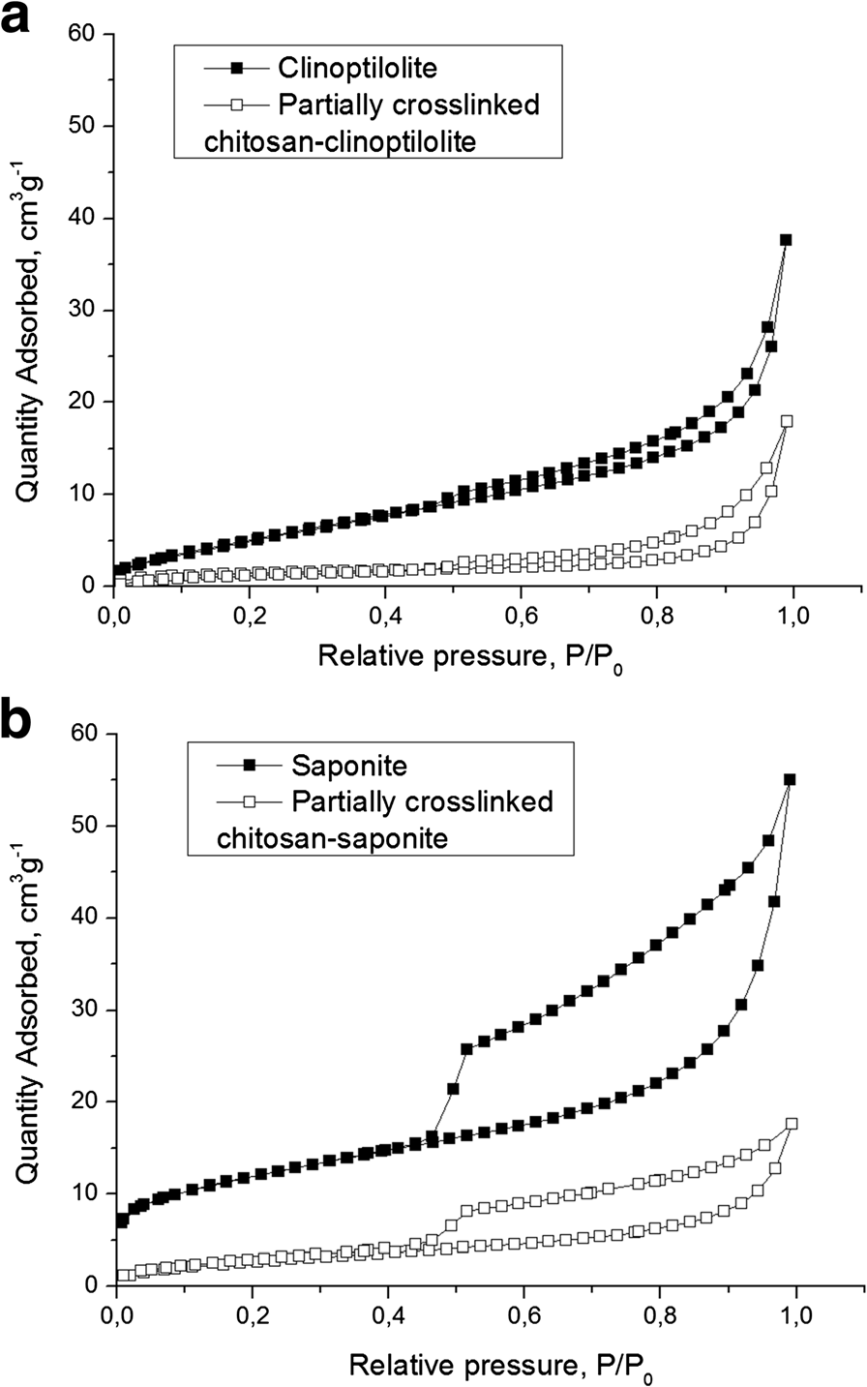
Nitrogen adsorption/desorption isotherms of the clinoptilolite and partially crosslinked chitosan-clinoptilolite (**a**) and saponite and partially crosslinked chitosan-saponite (**b**)

**Fig. 5:**
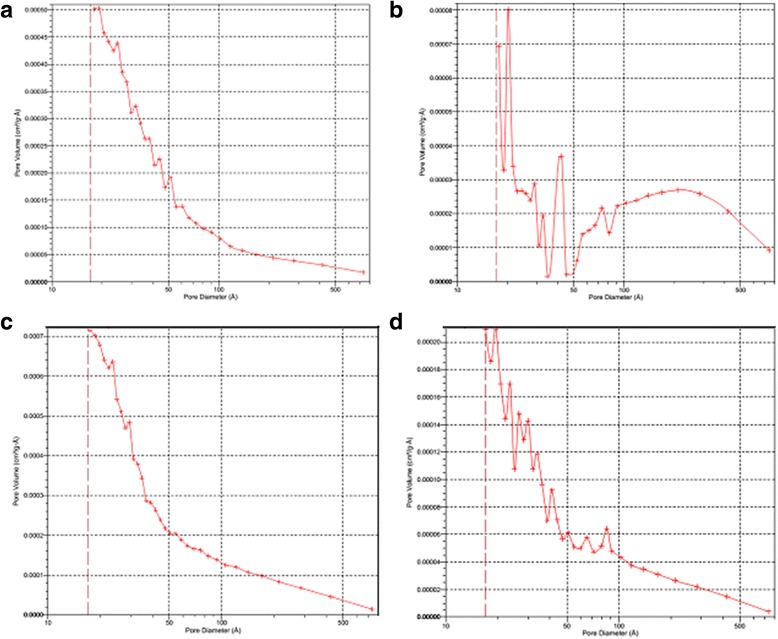
Pore-size distribution curve for the clinoptilolite (**a**), composite partially crosslinked chitosan-clinoptilolite (**b**), saponite (**c**), and composite partially crosslinked chitosan-saponite (**d**)

**Fig. 6:**
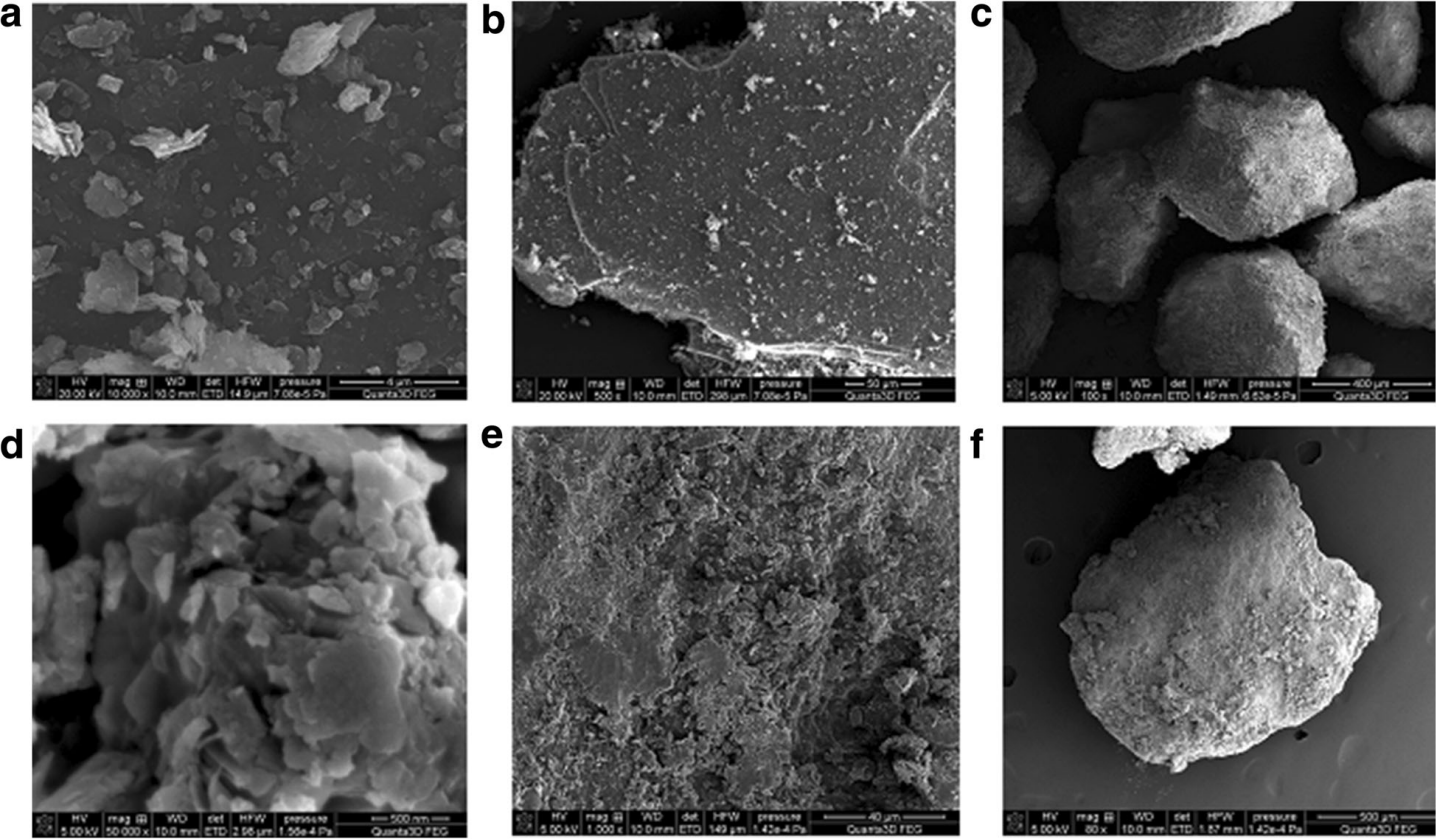
The SEM microphotographs of clinoptilolite at ×10,000 (**a**), ×500 (**b**), and ×100 (**c**) magnification and of partially crosslinked chitosan-clinoptilolite at ×50,000 (**d**), ×1000 (**e**), and ×100 (**f**) magnification

**Fig. 7:**
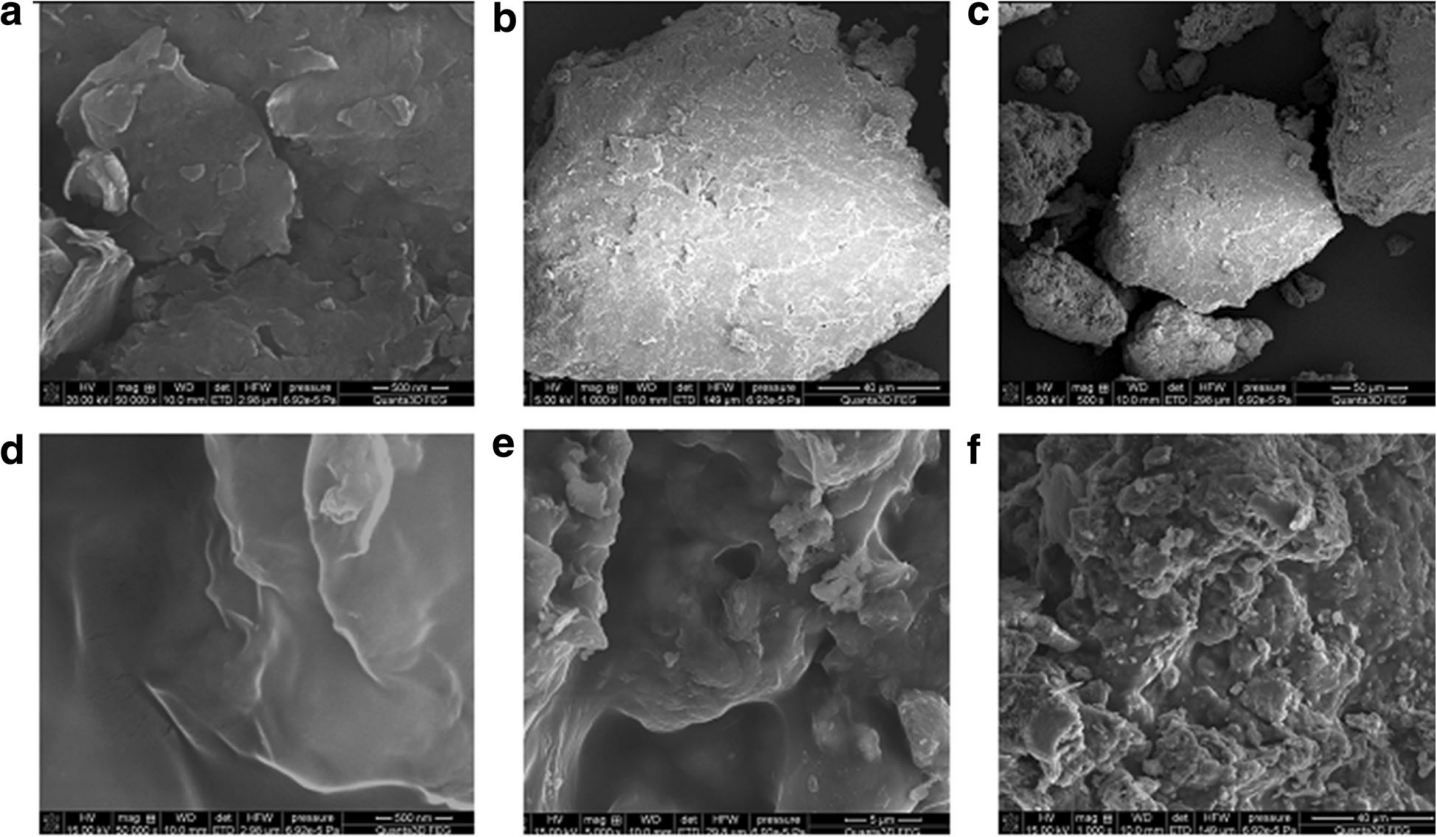
The SEM microphotographs of saponite at ×50,000 (**a**), ×1000 (**b**), and ×500 (**c**) magnification and of partially crosslinked chitosan-saponite at ×50,000 (**d**), ×5000 (**e**), and ×1000 (**f**) magnification

Figure 4 presents the nitrogen adsorption/desorption isotherms measured at 77 K for the initial minerals and coated minerals by chitosan. The shape of the isotherm corresponds to the Langmuir isotherm, type II of the International Union of Pure and Applied Chemistry (IUPAC) classification. This type of isotherm commonly observed in nonporous or macroporous materials of which the steep increase of adsorbed quantity at low relative pressure indicates the presence of unrestricted monolayer and multilayer adsorption. It is seen from the isotherms that the monolayer coverage completed at the relative pressure ranges up to 0.45. The shape of the isotherms confirms prevalent presence of cylindrical pores. According to the results of surface area analysis, the pure clinoptilolite and saponite has the BET surface area 22 and 41 m^2^/g, respectively, which was decreased with modification of its surfaces by polymer up to 5 and 10 m^2^/g for partially crosslinked chitosan-clinoptilolite and partially crosslinked chitosan-saponite. The presence of mesopores and macropores is confirmed by the diagram of pore size distribution for the initial and modified minerals (Fig. 5), which was obtained by the adsorption branch of the isotherm using the BJH method. The SEM images showed uniform coating of the surface of the minerals by chitosan (Figs. 6 and 7).

### Influence of pH on Adsorption

Montmorillonites and clays are perspective ion-exchangers; however, it is necessary to study the ability of those minerals to adsorb cationic forms of heavy metals, which is presented in natural waters and wastewaters. It is crucial to study factors which could influence the sorption behavior. For instance, the medium acidity is a very important factor because its plain main role on ionic form of the metals in aqueous solutions. In an acidic medium created by hydrochloric or acetic acid, metals such Cu(II), Zn(II), Fe(III), Cd(II), and Pb(II) could be present in a form of chlorides and acetates. Investigation of sorption properties of the synthesized composite began with the determination of medium acidity for the highest removal of the studied ions.

The degree of adsorption of Cu(II), Zn(II), Fe(III), Cd(II), and Pb(II) cations by composites based on partially crosslinked chitosan and natural minerals clinoptilolite and saponite as a function of the medium acidity were investigated in different chemical compositions of buffer solutions. Ionic forms of cations presented in Table 2. The obtained degree of adsorption of Cu(II), Zn(II), Fe(III), Cd(II), and Pb(II) cations by studied composites is presented in Table 3. It can be seen that the highest degree of adsorption (up to 99.00%) on the surface of the obtained composite was observed for all cations from the solutions with a concentration 4 mg/l of studied metals in the slightly basic (pH 8.0, ammonium acetate buffer) and neutral medium. In the acidic medium, the decreasing of degree of adsorption of cationic forms of studied metals was observed.

**Table 2:** Forms of existing cations of transition metals as a function of the medium acidity and chemical composition of buffer solution

Ion	pH				
	pH 1.0 HCl	pH 2.5 acetic acid	pH 5.0 acetate buffer	Distilled water	pH 8.0 ammonium acetate buffer
Zn(II)	[Zn(H_2_O)_6_]^2+^ [ZnCl_4_]^2−^	[Zn(CH_3_COO)_2_(H_2_O)_2_]^2−^ [Zn(H_2_O)_6_]^2+^	[Zn(CH_3_COO)_2_(H_2_O)_2_]^2−^ [Zn(H_2_O)_6_]^2+^	[Zn(H_2_O)_6_]^2+^	[Zn(CH_3_COO)_2_(H_2_O)_2_]^2−^ [Zn(NH_3_)_4_]^2+^ [Zn(NH_3_)_2_(CH_3_COO)_2_]
Cu(II)	[Cu(H_2_O)_6_]^2+^ [CuCl_4_]^2−^	[Cu_2_(CH_3_COO)_2_(H_2_O)_2_]^2+^ [Cu(H_2_O)_6_]^2+^	[Cu_2_(CH_3_COO)_2_(H_2_O)_2_]^2+^ [Cu(H_2_O)_6_]^2+^	[Cu(H_2_O)_6_]^2+^	[Cu_2_(CH_3_COO)_2_(H_2_O)_2_]^2+^ [Cu(NH_3_)_2_(H_2_O)_4_]^2+^ [Cu(NH_3_)_4_(H_2_O)_2_]^2+^
Cd(II)	[Cd(H_2_O)_4_]^2+^ [CdCl_4_]^2−^ [CdCl_3_]^−^	[Cd(CH_3_COO)_4_]^2−^ [Cd(H_2_O)_4_]^2+^	[Cd(CH_3_COO)_4_]^2−^ [Cd(H_2_O)_4_]^2+^	[Cd(H_2_O)_4_]^2+^	[Cd(NH_3_)_4_]^2+^ [Cd(CH_3_COO)_4_]^2−^ [Cd(NH_3_)(CH_3_COO)_3_] ^–^
Pb(II)	[PbCl_4_]^2−^ [PbCl_3_]^−^	[Pb(H_2_O)_6_]^2+^ [Pb(OH)(H_2_O)_2_(CH_3_COO)] ^2-^	[Pb(H_2_O)_6_]^2+^ [Pb(OH)(H_2_O)_2_(CH_3_COO)] ^2-^	[Pb(H_2_O)_6_]^2+^	[Pb(OH)(H_2_O)_3_]^+^ [Pb(OH)(H_2_O)_2_(CH_3_COO)] ^2-^
Fe(III)	[Fe(H_2_O)_6_]^3+^ [FeCl_2_]^+^ [FeCl_4_]^−^	[Fe(H_2_O)_6_]^3+^ [Fe_3_O(CH_3_COO)_6_](CH_3_COO)_2_	[Fe(H_2_O)_6_]^3+^ [Fe_3_O(CH_3_COO)_6_](CH_3_COO)_2_	[Fe(H_2_O)_6_]^3+^ [Fe(H_2_O)_5_(OH)]^2+^ [Fe(H_2_O)_4_(OH)_2_]^+^	[Fe_3_O(CH_3_COO)_6_](CH_3_COO)_2_ [Fe(NH_3_)_6_]^3+^

**Table 3:** The degree of adsorption of Zn(II), Cu(II), Fe(III), Cd(II), and Pb(II) cations by synthesized composites as a function of the medium acidity

Ions	Degree of adsorption (%)				
	pH 1.0 HCl	pH 2.5 acetic acid	pH 5.0 acetate buffer	Distilled water	pH 8.0 ammonium acetate buffer
Partially crosslinked chitosan-clinoptilolite composite					
Zn(II)	10.60	0.00	0.00	32.60	47.33
Cu(II)	0.00	2.5	10.00	65.25	86.00
Cd(II)	7.5	2.5	3.75	49.50	37.00
Pb(II)	27.00	47.00	13.75	97.75	99.00
Fe(III)	0.00	2.50	15.00	42.50	90.00
Partially crosslinked chitosan-saponite composite					
Zn(II)	1.33	2.33	0.00	35.33	69.66
Cu(II)	0.00	12.5	10.00	81.50	86.00
Cd(II)	7.00	2.5	3.75	34.50	28.50
Pb(II)	12.75	4.75	7.00	93.25	97.75
Fe(III)	0.00	2.50	20.00	70.20	92.50

Experimental conditions: mass of sorbent—0.1 g, volume of solution—20 ml, *m* ^0^_Zn_—60 μg, *m* ^0^_Fe, Cu, Cd, Pb_—80 μg

Thus, the synthesized composite showed adsorption activity with respect to the investigated ions in neutral and slightly basic medium and confirmed that the adsorption process occurs through complexation of aqua, acetic, or bi-ligand complexes of studied ions with amino groups of chitosan. The values of medium acidity, at which the maximum adsorption activities of chitosan-based composites for each of the studied ions were achieved, correspond to the published data of complexation conditions of these ions with amino groups of chitosan in solutions [28].

### Influence of Contact Time on Adsorption

According to the obtained results for all studied ions presented in Table 4, the degree of adsorption consistently increases for several hours, but the maximum degree of adsorption of all studied ions by the composites surface is achieved for a day which is typical of polymeric adsorbents where the sorption characteristics are defined by interactions between the ions and the functional groups of supported chitosan, which indirectly confirm that the adsorption process occurred by chitosan.

**Table 4:** Dependence of adsorption (*R*, %) on time of contact with the solutions containing 1 mg of molybdenum at pH 2.5, 1 mg of vanadium, and 200 μg of chromium in the neutral medium

Ions	Degree of adsorption (%)					
	5 min	10 min	30 min	60 min	90 min	Day
Partially crosslinked chitosan-clinoptilolite composite						
Zn(II)	46.00	46.25	46.25	46.50	46.50	32.60
Cu(II)	70.00	75.00	76.75	76.75	77.00	65.25
Cd(II)	46.90	48.70	49.60	49.90	49.90	49.50
Pb(II)	89.00	89.50	94.50	94.75	94.50	97.75
Fe(III)	39.00	35.00	35.00	35.25	35.25	42.50
Partially crosslinked chitosan-saponite composite						
Zn(II)	30.25	32.00	34.50	35.25	35.25	35.33
Cu(II)	72.50	77.00	77.50	80.20	80.75	81.50
Cd(II)	14.00	17.03	31.00	33.40	34.00	34.50
Pb(II)	73.25	73.50	73.75	73.75	73.75	93.25
Fe(III)	39.00	50.50	50.75	50.75	51.25	70.20

Experimental conditions: mass of sorbent—0.1 g, volume of solution—20 ml, *m* ^0^_Zn_—60 μg for chitosan-clinoptilolite, *m* ^0^_Zn_—60 μg for chitosan-saponite, *m* ^0^_Fe, Cu, Cd, Pb_—80 μg

### Influence of the Initial Metal Ion Concentration on Adsorption

**Fig. 8:**
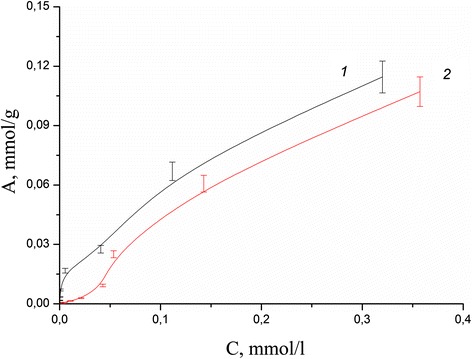
Adsorption isotherms of Cd(II) cations on the clinoptilolite (*1*) and partially crosslinked chitosan-clinoptilolite composite (*2*) in neutral media

**Fig. 9:**
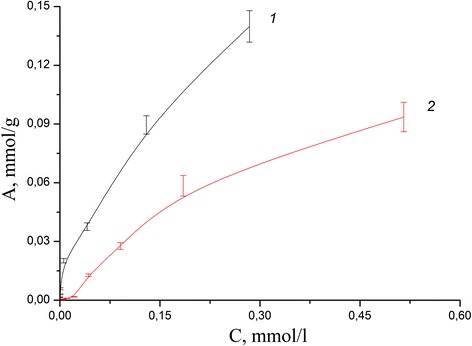
Adsorption isotherms of Cd(II) cations on the saponite (*1*) and partially crosslinked chitosan-saponite composite (*2*) in neutral media

**Fig. 10:**
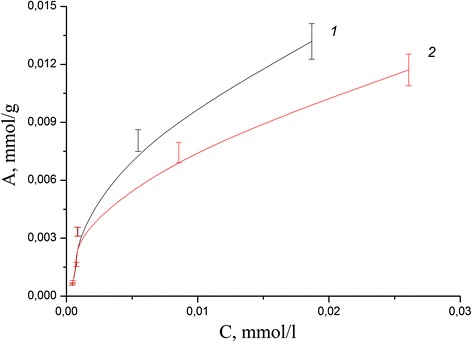
Adsorption isotherms of Pb(II) cations on the clinoptilolite (*1*) and partially crosslinked chitosan-clinoptilolite composite (*2*) in neutral media

**Fig. 11:**
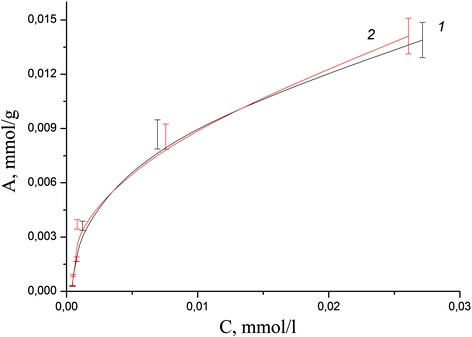
Adsorption isotherms of Pb(II) cations on the saponite (*1*) and partially crosslinked chitosan-saponite composite (*2*) in neutral media

Adsorption isotherms in the static mode for each ion were obtained for calculation of the values of the adsorption capacity of the composite and compare with the values for the initial minerals. The study of influence of initial metal ion concentration on adsorption in neutral medium has shown that adsorption capacity of coated clinoptilolite and saponite by chitosan in case of Pb(II) aqua ions reached more higher values than for pure minerals (Figs. 8, 9, 10, and 11). The values of adsorption capacity of chitosan-clinoptilolite and chitosan-saponite composites compared to pure minerals presented in Table 5. Obtained results has shown that the ability of chitosan to coordinate heavy metal ions Zn(II), Cu(II), Cd(II), and Fe(III) is less or equal to the ability to retain ions of these metals in the pores of minerals without forming chemical bonds.

**Table 5:** Adsorption capacity of chitosan-clinoptilolite and chitosan-saponite composites compared to a pure minerals

Ion	Adsorption capacity, mmol/g			
	Partially crosslinked chitosan-clinoptilolite composite	Clinoptilolite	Partially crosslinked chitosan-saponite composite	Saponite
Zn(II)	0.049	0.048	0.049	0.050
Cu(II)	0.115	0.113	0.131	0.129
Cd(II)	0.107	0.114	0.09	0.14
Pb(II)	0.013	0.012	0.014	0.014
Fe(III)	0.26	0.26	0.26	0.26

Experimental conditions: mass of sorbents—0.1 g, volume of solution—20 ml

## Conclusions

An investigation of properties of coated minerals of Ukrainian origin clinoptilolite and saponite by biopolymer chitosan has shown the number of advantages of obtained materials from the side of their physical-chemical properties. It was found that the synthesized composites contain the best characteristics of the initial materials: high biocompatibility and complexation ability of functional groups of chitosan and low cost and environmental friendliness of minerals clinoptilolite and saponite. The study of complexation properties of coated minerals by chitosan has shown that the ability of chitosan to coordinate heavy metal ions Zn(II), Cu(II), Cd(II), and Fe(III) is less or equal to the ability to retain ions of these metals in the pores of minerals without forming chemical bonds.
