# Erratum to: Natural Minerals Coated by Biopolymer Chitosan: Synthesis, Physicochemical, and Adsorption Properties

**DOI:** 10.1186/s11671-016-1752-7

**Published:** 2017-01-09

**Authors:** T. M. Budnyak, E. S. Yanovska, O. Yu. Kichkiruk, D. Sternik, V. A. Tertykh

**Affiliations:** 1 Chuiko Institute of Surface Chemistry of National Academy of Sciences of Ukraine, 17 General Naumov Str, 03164 Kyiv, Ukraine; 2 Taras Shevchenko National University of Kyiv, 62a Volodymyrska Str, 01033 Kyiv, Ukraine; 3 Zhytomyr Ivan Franko State University, 42 Pushkina Str, Zhytomyr, Ukraine; 4 Maria Curie-Skłodowska University, 2 Maria Curie Sklodowska Sq, 20-031 Lublin, Poland

## Erratum

Unfortunately [1], the title for Table 3 in the original version of this paper was incorrect.

The correct title for Table three is ‘The degree of adsorption of Zn(II), Cu(II), Fe(III), Cd(II), and Pb(II) cations by synthesized composites as a function of the medium acidity’.
